# Guy’s Hospital Dental School

**Published:** 1890-05

**Authors:** 


### Guy's Hospital Dental School.

A Lectureship on Operative Dental Surgery has been founded, and Mr. Harrold Murray has been appointed to the chair. The lectures will be delivered yearly during the Summer Session. The course will include the methods of examining the mouth for dental lesions, the exclusion of saliva during operations, the preparation of typical cavities for the reception of gold and other fillings. The lectures will deal with the scientific principles involved in the various methods of inserting the fillings now in use, individually or in combination with one another. Special attention will be directed to the treatment of pulpless teeth, and exposed pulps. The operative treatment of "Riggs' disease," the preparation of roots for pivots, the inlaying of teeth with porcelain, and the application of splints to fractured jaws will be fully discussed.

